# Multivariate Analysis of the Ocular Response Analyzer's Corneal Deformation Response Curve for Early Keratoconus Detection

**DOI:** 10.1155/2015/496382

**Published:** 2015-05-14

**Authors:** Jonatán D. Galletti, Pablo R. Ruiseñor Vázquez, Fernando Fuentes Bonthoux, Tomás Pförtner, Jeremías G. Galletti

**Affiliations:** ^1^ECOS (Clinical Ocular Studies) Laboratory, Pueyrredón 1716, 1119 Buenos Aires, Argentina; ^2^Institute of Experimental Medicine, National Academy of Medicine/CONICET, Pacheco de Melo 3081, 1425 Buenos Aires, Argentina

## Abstract

*Purpose*. To thoroughly analyze corneal deformation responses curves obtained by Ocular Response Analyzer (ORA) testing in order to improve subclinical keratoconus detection. *Methods*. Observational case series of 87 control and 73 subclinical keratoconus eyes. Examination included corneal topography, tomography, and biomechanical testing with ORA. Factor analysis, logistic regression, and receiver operating characteristic curves were used to extract combinations of 45 corneal waveform descriptors. Main outcome measures were corneal-thickness-corrected corneal resistance factor (ccCRF), combinations of corneal descriptors, and their diagnostic performance. *Results*. Thirty-seven descriptors differed significantly in means between groups, and among them ccCRF afforded the highest individual diagnostic performance. Factor analysis identified first- and second-peak related descriptors as the most variable one. However, conventional biomechanical descriptors corneal resistance factor and hysteresis differed the most between control and keratoconic eyes. A combination of three factors including several corneal descriptors did not show better diagnostic performance than a combination of conventional indices. *Conclusion*. Multivariate analysis of ORA signals did not surpass simpler models in subclinical keratoconus detection, and there is considerable overlap between normal and ectatic eyes irrespective of the analysis model. Conventional biomechanical indices seem to already provide the best performance when appropriately considered.

## 1. Introduction

The release of the Ocular Response Analyzer (ORA) device in 2005 made corneal biomechanical testing clinically possible [1], and many potential uses for this new technology have since been proposed. Among them, early detection of keratoconus and of corneas at risk of developing post-refractive-surgery ectasia is perhaps the most widely explored application in the literature [2–5]. The ORA brought along new corneal indices that attempted to describe the deformation response curve to a controlled air puff, which can readily be obtained from the software: corneal hysteresis (CH) and corneal resistance factor (CRF) [6, 7]. As research progressed, it soon became evident that there were limitations to the ORA's approach [6, 7] or, in other words, that the measured deformation response involved additional corneal properties besides the actual tissue's biomechanical properties. This was particularly the case for the device's low ability for keratoconus diagnosis that was initially reported [2, 8]. Insufficient knowledge of the many factors that affect corneal biomechanical testing led investigators to simply compare CH and CRF between control subjects and keratoconic patients and thus conclude that they performed poorly at disease detection. However, more recently we have shown that acceptable diagnostic capacity can be obtained by correcting those measurements for corneal thickness [3], one of the most influential factors, and that the results can be further improved by combining corneal compensated CRF and the difference between CH and CRF [5].

The ORA's corneal deformation waveform is a complex signal that stores considerably more information than just the interrelation of the inward and outward applanation pressure conveyed by CH and CRF [9]. The ORA software provides 37 additional descriptors that further describe each signal [4], but this information is only saved in the database and not reported to the user. An additional software module allegedly analyzes these waveform parameters, but the methodology behind the calculations was not disclosed by the ORA's manufacturer, thus preventing proper independent validation [10, 11]. The prospect of improved keratoconus detection by multivariate waveform analysis is attractive [4, 12, 13], but some considerations should be taken into account before additional data is to be extracted. Multicollinearity, the potential intercorrelation between the multiple parameters that are included in the predictive model can preclude the identification of the most appropriate ones [14]. There are, however, statistical tools that address this issue by extracting the most significant information from a group of interrelated variables. We hypothesized that comprehensive multivariate mining of ORA waveforms would yield additional information that might improve keratoconus detection. We therefore set out to analyze ORA measurements from nonkeratoconic and keratoconic patients in order to identify the most useful predictors and to compare multivariate models with already validated, simpler models.

## 2. Materials and Methods

The study was an observational case series. The research protocol followed the tenets of the Declaration of Helsinki and was approved by an ethics committee. All subjects were told of the purpose of the study and gave written informed consent before inclusion. Patients were recruited between March 2010 and November 2011 at ECOS Laboratory and had been referred for spectacle or contact lens prescription or keratoconus diagnosis. Each subject underwent slit-lamp examination, anterior segment optical coherence tomography (OCT) evaluation (software version 2.0.1.88, Visante OCT, Carl Zeiss Meditec, Dublin, CA, USA), Placido disk topography and aberrometry (software version 4.0, iTrace, Tracey Technologies, Houston, TX, USA), and ORA measurements (software version 2.04, Reichert Ophthalmic Instruments, Depew, NY, USA). All patients were examined by two trained ophthalmologists between 1 PM and 6 PM. Topographic exams with artifacts were discarded. Topographic indices such as average corneal power and higher-order aberrations of the corneal 5 mm central surface were provided by iTrace software. Central corneal thickness was obtained from the mean value of the 2 mm central area of the OCT Visante pachymetry map.

For topography and keratoconus grading, the keratoconus severity score (KSS) was used [15], which is based on average corneal power (in diopters) and corneal higher-order aberrations (HOA, expressed in *μ*m as root-mean-square values). The KSS scale ranges between 0 (unaffected, normal topography), 1 (unaffected, atypical topography), 2 (suspect), 3 (mild keratoconus), 4 (moderate keratoconus), and 5 (severe keratoconus). Subjects (one eye chosen at random) were included in group 1 if they showed no clinical signs of ectasia and KSS 0 (unremarkable topography defined as typical axial pattern and higher-order aberrations < 0.65 *μ*m) in both eyes. Such strict topographical criteria for nonkeratoconic subjects were adapted from Buhren et al. [16], as some subclinical keratoconus cases initially show increased higher-order aberrations. Eyes with subclinical keratoconus (group 2) were defined following the criteria of Buhren et al. [16], that is, eyes with no clinical signs of ectasia and KSS 0, 1, or 2 from patients with manifest keratoconus (KSS 3 or higher) in the fellow eye: axial topography pattern consistent with keratoconus may have positive slit-lamp findings, no corneal scarring, average corneal power > 49.00 D, and higher-order aberrations > 1.50 *μ*m. Participant exclusion criteria were the following: age less than 18 years, previous eye surgery, any eye disease other than keratoconus, and chronic use of topical medications or corneal opacities. Contact lenses were removed at least 72 hours before examination.

For corneal biomechanical testing, four consecutive ORA measurements without topical anesthesia were obtained and averaged (only good-quality readings, as defined by the manufacturer, were stored). The details of the ORA function and the applanation pressures from which both CH and CRF are derived have been addressed elsewhere [17]. The 37 built-in waveform descriptors were extracted from the ORA database, and temporal descriptors were computed as described by Mikielewicz et al. [4]. The methodology for calculating central-corneal-thickness-corrected CH (ccCH) and CRF (ccCRF) was reported elsewhere [9] and that for CH-CRF was published by Touboul et al. [18]. The combination of ccCRF and CH-CRF through logistic regression, termed biomechanical score (BiomechScore), has also been thoroughly described [5]. Factor analysis was performed on all biomechanical descriptors from both groups combined with principal components analysis as extraction method and varimax as rotation method. Seven factors with eigenvalues > 1 were extracted and the actual solution was corroborated with a scree plot. Factors were named according to the observed patterns in variable loadings. Logistic regression was then used to combine the three factors (3FactorScore) that showed statistically significant differences in factor scores between the two groups. It should be noted that this logistic regression analysis was performed on the factor scores and therefore is akin to principal components regression in that it is not affected by multicollinearity of the covariates. Receiver-operating characteristic (ROC) curves were used to calculate sensitivity, specificity, and area under the curve (AROC) of each biomechanical descriptor taken separately and of each logistic function. Statistical analysis was performed with SPSS 17 software (SPSS Inc., Chicago, IL, USA). Normality of every variable was determined by the Kolmogorov-Smirnov test and parametric or nonparametric tests were used accordingly. Statistical significance was set at *p* < 0.05 and data are shown as mean ± standard deviation unless otherwise stated. Data collection and sorting were done with the aid of Microsoft Excel 2010 software.

## 3. Results

### 3.1. Demographics

Demographics and corneal characteristics of control and keratoconic eyes are summarized in Table 1. All control cases had unremarkable topography (KSS 0), whereas, in the keratoconus group, 45 (62%) were KSS 0, 15 (20%) were KSS 1, and 13 (18%) were KSS 2 in topography grading.

### 3.2. Individual Waveform Descriptors and Previously Described Indices

All 45 biomechanical descriptors were normally distributed in both groups. In control eyes, central corneal thickness was significantly correlated with CH, CRF, timein, timeout, bindex, p1area, w2, path1, p1area1, w11, w21, and path11. In keratoconic eyes, central corneal thickness was significantly correlated with CH, CRF, timein, timeout, p1area, p2area, path1, p1area1, p2area1, w11, path11, and path21. Thirty-seven biomechanical descriptors differed significantly in means between control and keratoconic eyes (Table 2).

**Figure 1:**
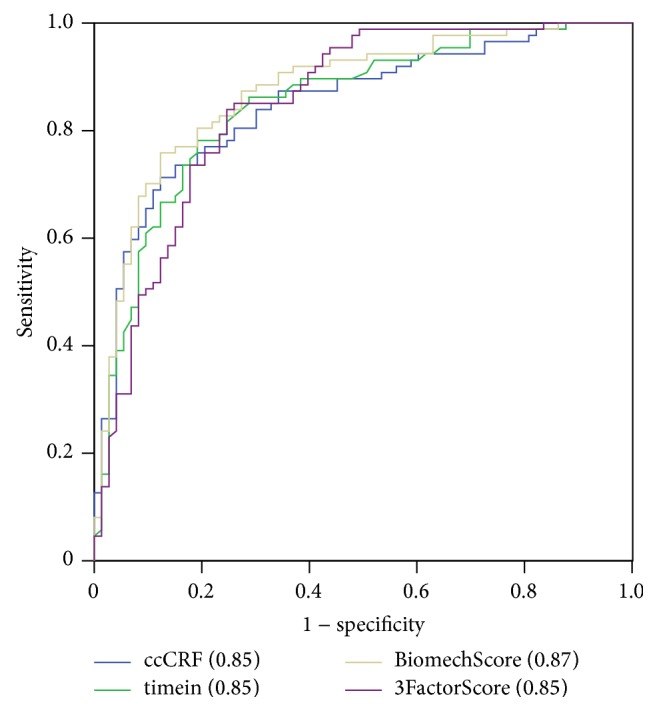
Comparative diagnostic capacity of corneal biomechanical indices. Receiver-operating characteristic curves are plotted for central-corneal-thickness-corrected corneal resistance factor (ccCRF), timein, a combined index that includes ccCRF (BiomechScore), and the combination of the three extracted factors (3FactorScore). See Methods for details on each index. Area under the curve is specified in parentheses for each index.

With respect to diagnostic capacity (Table 3 and Figure 1), ccCRF ranked highest amongst the individual descriptors, with 71.3% specificity, 86.7% sensitivity, and an area under the curve of 0.85 (95% CI 0.79–0.91). The previously described combination of ccCRF and CH-CRF (biomechanical score (BiomechScore)) showed 81.6% specificity and 76.7% sensitivity, with an area under the curve of 0.87 (95% CI 0.82–0.93). The specificity and sensitivity of the optimum cutoff for the 12 best individual biomechanical descriptors are summarized in Table 3. The specified cutoffs were used to calculate a dichotomous “normal” or “abnormal” value for each case, and then the number of abnormal descriptors was counted for each observation to yield a descriptor score (9DescScore). In order to reduce multicollinearity, only the better performing descriptor of the pairs concerning the same aspects of the waveform (ccCRF and CRF, h2 and h21, and p2area1 and p2area) was considered, which led to a total number of 9 descriptors in the score (CRF, h21, and p2area were excluded). The 9DescScore had 90.8% specificity and 74.0% sensitivity with an optimal cutoff of >4 and an area under the curve of 0.89 (95% CI 0.84–0.94). Forward stepwise logistic regression of these 9 individual dichotomous variables resulted in a similar error rate (area under the curve 0.90, 95% CI 0.85–0.94) by including only 4 descriptors (ccCRF, CH-CRF, h2, and dive 2) and a more balanced performance in the control and keratoconic groups: 85.1% specificity and 78.1% sensitivity.

### 3.3. Multivariate Analysis of the ORA Waveform

A combination of waveform descriptors could have better diagnostic performance that the individual variables considered alone, but correlation between descriptors (multicollinearity) negatively affects multivariate classification models. In order to correct for this situation, principal component analysis was performed first on 37 descriptors from both groups combined to identify the main underlying factors (groups of descriptors that vary altogether, indicating that they measure similar aspects of the waveform). Eight descriptors had to be excluded from the analysis because they were completely determined by others and therefore were perfectly intercorrelated (e.g., CH-CRF was totally dependent on CH and CRF, deltatime on timein and timeout, h11 on h1, and h2 on h21).

The seven factors extracted amounted to 86.3% of total variance and are summarized in Table 4. They were named according to the waveform descriptors on which they were extracted. Factor scores for each observation were computed and compared between groups. Only factors Peak2 (*p* < 0.001), Peak1 (*p* = 0.01), and Conventional (*p* < 0.001) differed significantly in means between control and keratoconic eyes, with areas under the curve of 0.67, 0.62, and 0.78, respectively. Logistic regression analysis was used to combine these three factors into a diagnostic function (termed 3FactorScore), attaining 83.9% specificity and 74.0% sensitivity, with an area under the curve of 0.85 (95% CI 0.79–0.91).

### 3.4. Comparison of the Best Diagnostic Indices

**Figure 2:**
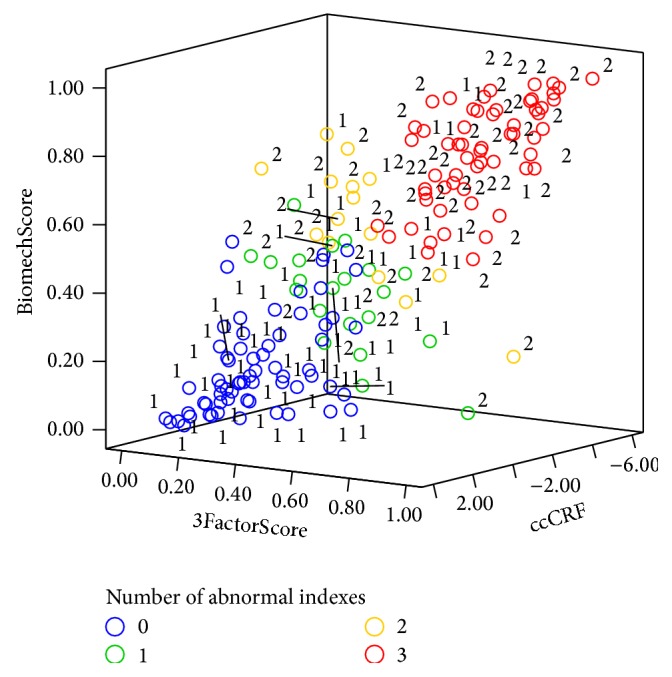
Distribution of the three best biomechanical indices for both groups combined. Three-dimensional plot of ccCRF, BiomechScore, and 3FactorScore in which the color of each observation indicates the number of abnormal indices (from 0 to 3). The number next to each observation indicates the group to which it belongs (group 1 or 2).

**Figure 3:**
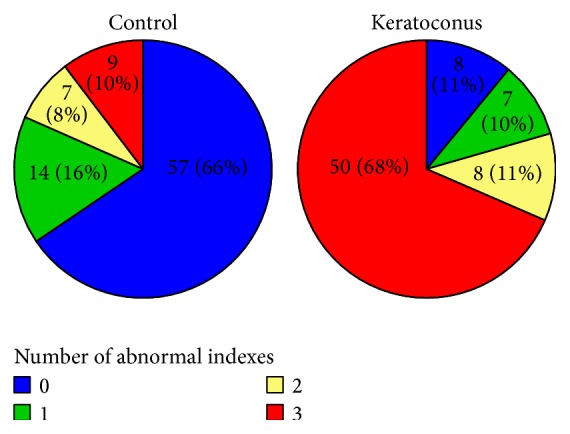
Group composition by number of abnormal biomechanical indices. Groups 1 (control) and 2 (subclinical keratoconus) represented in pie charts by the number of abnormal biomechanical indices (ccCRF, BiomechScore, and 3FactorScore).

The indices ccCRF, BiomechScore, and 3FactorScore were significantly correlated between themselves (Figure 2). One-hundred twenty-four (77.5%) cases were concordant for the three indices, of which 65 (40.6%) showed normal values and 59 (36.9%) showed abnormal values. Twenty-one (13.1%) cases had only one abnormal index, of which 14 (8.8%) had abnormal ccCRF values and 2 (1.3%) and 5 (3.1%) had abnormal BiomechScore and 3FactorScore values, respectively. Fifteen cases showed two abnormal indices, all of which had abnormal ccCRF values and 11 (6.9%) and 4 (2.5%) had abnormal BiomechScore and 3FactorScore values, respectively. Within-group results are summarized in Figure 3. In the control group, 71 (81.6%) cases had up to one abnormal index and, in the keratoconus group, 58 (79.5%) cases had two or more abnormal indices. The area under the curve for the number of abnormal indices was 0.85 (95% CI 0.79–0.91), with an optimal cutoff of ≥2.

## 4. Discussion

In this work, we assessed additional information extracted from the corneal deformation response curves for their capacity to differentiate between control and keratoconic eyes. The actual analysis of the waveform signals was taken directly from the ORA software, which provides 37 descriptors in addition to CH and CRF. Mikielewicz et al. [4] have already described the meaning of these indices, and we refer to their work to avoid repetition. They also analyzed the diagnostic capacity of each descriptor individually and found that the indices describing the second peak in the ORA signal had excellent performance at diagnosing keratoconus (area under the curve >0.95). This conclusion, however, was based on comparing keratoconic eyes that were deemed treatable by either corneal crosslinking or intrastromal segments and therefore must have had other clinical or topographical signs of ectatic disease. But the actual usefulness of corneal biomechanical testing lies in the detection of early keratoconus cases, either with little topographical abnormalities or none at all. In this study we only included fellow eyes of verified keratoconus patients that had insufficient clinical and topographical findings to be regarded as ectatic when considered alone, as it is accepted that keratoconus is always a bilateral albeit asymmetric condition [19–21].

In our sample, only one of the additional corneal descriptors (timein) performed comparably to the corneal-thickness-corrected CRF, a modified version of this conventional biomechanical index that accounts for the sizable influence of corneal thickness on ORA measurements [3]. The actual performance for both indices was remarkably lower than that reported by Mikielewicz et al. [4], and this difference reflects the subclinical nature of our keratoconic eyes. A combined score (BiomechScore) that considered both corneal-thickness-corrected CRF and the difference between CH and CRF (CH-CRF) had slightly better results, as we have previously shown [5]. Such approach works better when dealing with myopic eyes, which also have reduced CRF values (just as keratoconic eyes) but markedly negative CH-CRF values (keratoconic eyes have positive CH-CRF values). The alternative strategy of counting the number of abnormal descriptors that were previously selected by their better individual performance, termed 9DescScore, led to slightly better diagnostic performance in this study, but the difference may not be clinically meaningful and must be validated with independent samples.

In-depth analysis of the waveform descriptors showed that many were highly intercorrelated, which was expected as the manufacturer put out two sets of variables that refer to either the 75% or 50% height of the two peaks in the ORA signal [4]. In other words, the variables describe overlapping aspects of the waveform. Factor analysis, a statistical tool that identifies variation patterns in datasets with multiple variables, extracted seven independent factors. Factors correspond to groupings of variables in different proportions, in such a way to account for the most variance in the data. The first factor extracted grouped descriptors that refer to the second peak in the ORA signal, and this is in agreement with clinical experience with the device: the second peak is the one that varies the most between subjects. We therefore named this factor Peak2. Then a comparable grouping was extracted for the first peak, which we named Peak1. The two factors explained almost half of the total variance. Third, a more limited group of descriptors that applied to the first peak again was obtained, but this time only the more basic aspects were included: height, width, and enclosed area. Thus, the factor was termed OverallPeak1. The fourth factor included similar aspects of the second peak, which was named accordingly OverallPeak2. Remarkably, the conventional biomechanical indices CH and CRF were extracted combined as the fifth factor, thus named Conventional. The sixth factor, which amounted to only 4.1% of the total variance, included a few descriptors from the first and second peaks and the area between the two. We named it OverallWaveform. The seventh and last factor (Peak1Extra) contained only one variable (aindex) that describes the roughness of the first peak.

The seven extracted factors allowed for a simpler perspective of the complex ORA signal. Only factors Peak2, Peak1, and Conventional differed significantly between control and keratoconic eyes, suggesting that the variability in the other four was mainly due to differences between cases and not between groups. Despite being the fifth factor in explained variance, factor Conventional exhibited the largest mean difference between control and keratoconic eyes, surpassing the more variable factors Peak2 and Peak1 in their diagnostic ability for ectatic corneas. In other words, multivariate analysis suggested that the conventional CH and CRF are the most appropriate indicators of an ectatic biomechanical profile. Supporting this conclusion, a multivariate diagnostic model (3FactorScore) that included the three meaningful factors Peak2, Peak 1, and Conventional was slightly inferior to the previously reported BiomechScore in detecting keratoconic eyes (Figure 1). Moreover, another multivariate diagnostic model that allowed for free inclusion of the 9 best individual descriptors only selected corneal-thickness-corrected CRF, CH-CRF, h2, and dive2 in descending order of importance on the outcome.

In summary, this study shows that, irrespective of the analysis performed on the ORA signal, there seems to be no complete separation between control and subclinical keratoconus eyes (Figure 2). This could be due to limitations in the sensitivity of the device or perhaps indicate that certain fellow eyes of overt keratoconus patients at the time they were examined did not have any biomechanical abnormalities. It has been recently put forth that there is a biomechanical cycle of decompensation in corneal ectasia and that the biomechanical disruption could be focal at first instead of generalized [22]. Our data suggests that an abnormal ORA waveform response is not observed in every fellow eye of keratoconus patients, which could indicate that some of those corneas were measured at an earlier stage in the decompensation cycle or perhaps outside of the focal abnormality. It has been argued that additional analysis of the ORA signal could yield additional information [4], but our work suggests otherwise. It may well be the case that there is no distinct biomechanical signature at the earliest stage in keratoconus progression.

## 5. Conclusion

Our work shows that multiparametric analysis of ORA's waveform signals does not increase the diagnostic yield for keratoconic corneas. It also serves as further validation of the previously proposed combinations of the conventional biomechanical indices, which seem to already provide the highest sensitivity and specificity for subclinical ectasia. Although the attained diagnostic performance is far from perfect, it is considerably better than that afforded by other methods for truly subclinical keratoconic eyes [23].

## Figures and Tables

**Table 1:** Demographics and corneal characteristics of control and keratoconic eyes. Demographics and corneal characteristics of control (group 1) and subclinical keratoconus (group 2) eyes. Data is expressed as mean ± standard deviation.

	Group 1 (*n* = 87)	Group 2 (*n* = 73)	*p*
Age (years)	35 ± 12	34 ± 11	0.429
Gender (male/female)	30/57	46/27	**<0.001**
Average corneal power (D)	43.74 ± 1.65	44.25 ± 2.04	0.078
Central corneal thickness (*µ*m)	513 ± 30	498 ± 31	**0.002**
Higher-order aberrations (*µ*m)	0.229 ± 0.083	0.586 ± 0.382	**<0.001**
Vertical comma (*µ*m)	0.082 ± 0.075	0.384 ± 0.349	**<0.001**
Horizontal comma (*µ*m)	0.069 ± 0.072	0.190 ± 0.210	**0.001**

**Table 2:** Biomechanical descriptors in control and keratoconic eyes. Corneal waveform descriptors of control (group 1) and subclinical keratoconus (group 2) eyes. Data is expressed as mean ± standard deviation. AROC = area under the curve; CI = confidence interval.

Descriptor	Group 1 (mean ± SD)	Group 2 (mean ± SD)	*p*	AROC (95% CI)
CH	9.62 ± 1.46	8.50 ± 1.36	**<0.001**	**0.71 (0.63–0.79)**
CRF	9.35 ± 1.46	7.37 ± 1.37	**<0.001**	**0.84 (0.78–0.9)**
ccCH	−0.17 ± 1.30	−1.04 ± 1.19	**<0.001**	**0.70 (0.62–0.78)**
ccCRF	−0.20 ± 1.22	−1.87 ± 1.13	**<0.001**	**0.85 (0.79–0.91)**
CH-CRF	0.27 ± 0.92	1.13 ± 0.78	**<0.001**	**0.77 (0.84–0.7)**
timein	7.74 ± 0.39	7.19 ± 0.37	**<0.001**	**0.85 (0.79–0.91)**
timeout	18.48 ± 0.23	18.35 ± 0.24	**0.001**	**0.65 (0.57–0.74)**
deltatime	10.74 ± 0.44	11.16 ± 0.45	**<0.001**	**0.76 (0.69–0.84)**
aindex	7.82 ± 1.14	7.27 ± 1.41	**0.008**	**0.62 (0.53–0.71)**
bindex	8.16 ± 1.46	6.98 ± 1.87	**<0.001**	**0.68 (0.6–0.77)**
p1area	3887.14 ± 867.29	3360.05 ± 1279.24	**0.003**	**0.68 (0.59–0.76)**
p2area	2237.06 ± 626.53	1787.33 ± 609.35	**<0.001**	**0.7 (0.62–0.78)**
aspect1	20.91 ± 4.59	18.39 ± 5.35	**0.002**	**0.64 (0.56–0.73)**
aspect2	18.57 ± 7.34	13.73 ± 7.29	**<0.001**	**0.69 (0.61–0.78)**
uslope1	79.80 ± 22.53	69.10 ± 22.13	**0.003**	**0.64 (0.55–0.73)**
uslope2	87.20 ± 32.35	66.82 ± 34.67	**<0.001**	**0.69 (0.6–0.77)**
dslope1	29.72 ± 6.93	26.63 ± 8.57	**0.014**	**0.62 (0.53–0.71)**
dslope2	24.38 ± 10.35	17.94 ± 9.71	**<0.001**	**0.68 (0.6–0.77)**
w1	22.30 ± 2.39	22.57 ± 2.84	0.513	0.54 (0.45–0.63)
w2	20.35 ± 4.81	22.51 ± 5.87	**0.013**	**0.61 (0.52–0.70)**
h1	440.97 ± 84.47	386.16 ± 107.79	**0.001**	**0.66 (0.57–0.75)**
h2	310.47 ± 83.16	235.14 ± 87.32	**<0.001**	**0.74 (0.66–0.82)**
dive1	367.21 ± 96.01	318.10 ± 99.11	**0.002**	**0.65 (0.56–0.74)**
dive2	234.06 ± 70.57	173.78 ± 71.64	**<0.001**	**0.74 (0.66–0.81)**
path1	24.64 ± 3.66	26.02 ± 4.50	**0.038**	**0.6 (0.69–0.51)**
path2	27.18 ± 4.86	27.44 ± 4.61	0.733	0.52 (0.43–0.61)
mslew1	141.48 ± 31.79	128.10 ± 32.48	**0.010**	**0.61 (0.53–0.7)**
mslew2	138.38 ± 43.12	106.51 ± 41.92	**<0.001**	**0.7 (0.62–0.78)**
slew1	85.83 ± 23.30	77.59 ± 22.41	**0.024**	**0.62 (0.53–0.71)**
slew2	89.07 ± 31.61	70.48 ± 33.82	**<0.001**	**0.68 (0.59–0.76)**
aplhf	1.63 ± 0.33	1.73 ± 0.38	0.105	0.57 (0.65–0.48)
p1area1	1612.69 ± 416.66	1381.53 ± 625.19	**0.008**	**0.67 (0.59–0.76)**
p2area1	937.39 ± 278.33	736.97 ± 290.89	**<0.001**	**0.71 (0.62–0.79)**
aspect11	30.77 ± 8.38	28.03 ± 9.16	0.052	**0.6 (0.51–0.69)**
aspect21	27.31 ± 10.44	20.31 ± 10.37	**<0.001**	**0.7 (0.62–0.78)**
uslope11	78.81 ± 22.38	69.05 ± 21.91	**0.006**	**0.64 (0.55–0.72)**
uslope21	71.29 ± 26.17	55.64 ± 28.37	**<0.001**	**0.68 (0.6–0.76)**
dslope11	51.57 ± 16.01	48.63 ± 17.63	0.276	0.55 (0.46–0.64)
dslope21	42.30 ± 17.25	30.58 ± 16.47	**<0.001**	**0.70 (0.62–0.78)**
w11	10.98 ± 2.06	10.71 ± 1.90	0.400	0.53 (0.44–0.62)
w21	9.31 ± 2.33	10.26 ± 2.88	**0.024**	**0.6 (0.51–0.69)**
h11	293.98 ± 56.31	257.44 ± 71.86	**0.001**	**0.66 (0.57–0.75)**
h21	206.98 ± 55.44	156.76 ± 58.22	**<0.001**	**0.74 (0.66–0.82)**
path11	35.76 ± 5.52	37.56 ± 7.06	0.078	0.59 (0.68–0.5)
path21	37.49 ± 6.59	37.87 ± 6.75	0.715	0.51 (0.6–0.42)

**Table 3:** Diagnostic capacity of individual descriptors. Specificity, sensitivity, and cutoff value for diagnosing subclinical keratoconus are shown for the 12 corneal waveform descriptors with the highest area under the curve in decreasing order.

	ccCRF	timein	CRF	CH-CRF	delta-time	h2	h21	dive2	CH	p2-area1	mslew2	p2-area
Spec	73.6	78.2	72.4	74.7	71.3	62.1	62.1	67.8	65.5	70.1	77.0	66.7
Sens	84.9	80.8	80.8	74.0	75.3	74.0	74.0	69.9	72.6	64.4	57.5	65.8
Cut-off	<−0.77	<7.44	<8.46	>0.85	>10.95	<277.8	<185	<205.8	<9.19	<817.6	<111.5	<1968.0

**Table 4:** Factor analysis of the corneal deformation response curve. Factors are shown on top in the order in which they were extracted (as described in Section 2). Corneal descriptor loadings are summarized for each factor.

Factor	1	2	3	4	5	6	7
Name	Peak2	Peak1	OverallPeak1	OverallPeak2	Conventional	OverallWaveform	Peak1Extra
% of Variance	27.7	22.0	12.1	8.7	7.8	4.1	3.8

Cumulative %	27.7	49.8	61.9	70.6	78.3	82.5	86.3

CH					0.907		
CRF					0.919		
timeout					0.801		
timein					0.618	0.504	
aplhf						0.671	
bindex	0.656					−0.531	
mslew2	0.945						
aspect2	0.944						
slew2	0.938						
aspect21	0.937						
uslope2	0.933						
h2	0.905						
dslope2	0.905						
dslope21	0.893						
uslope21	0.848						
dive2	0.832						
w2	−0.691						
p2area1	0.539			0.740			
p2area	0.512			0.744			
w21	−0.594			0.663			
path2				−0.790			
path21				−0.746			
uslope11		0.839					
uslope1		0.881					
slew1		0.892					
mslew1		0.901					
dslope1		0.790					
dslope11		0.727					
aspect1		0.897					
h1		0.825					
dive1		0.829					
aspect11		0.875					
p1area		0.511	0.780				
path11			−0.865				
p1area1			0.813				
path1			−0.917				
w11			0.801				
w1			0.599				
aindex							0.681
